# Is simulation training effective in increasing podiatrists' confidence in foot ulcer management?

**DOI:** 10.1186/1757-1146-4-16

**Published:** 2011-06-05

**Authors:** Peter A Lazzarini, Elizabeth L Mackenroth, Patricia M Régo, Frances M Boyle, Scott Jen, Ewan M Kinnear, Graham M PerryHaines, Maarten Kamp

**Affiliations:** 1 Allied Health Research Collaborative, Metro North Health Service District, Queensland Health, Australia; 2 Department of Podiatry, Metro North Health Service District, Queensland Health, Australia; 3 School of Public Health, Queensland University of Technology, Australia; 4 School of Medicine, The University of Queensland, Australia; 5 Clinical Skills Development Service, Centre for Healthcare Improvement, Queensland Health, Australia; 6 School of Population Health, The University of Queensland, Australia; 7 Department of Podiatry, Darling Downs-West Moreton Health Service District, Queensland Health, Australia; 8 Department of Endocrinology, Metro North Health Service District, Queensland Health, Australia

## Abstract

**Background:**

Foot ulcers are a frequent reason for diabetes-related hospitalisation. Clinical training is known to have a beneficial impact on foot ulcer outcomes. Clinical training using simulation techniques has rarely been used in the management of diabetes-related foot complications or chronic wounds. Simulation can be defined as a device or environment that attempts to replicate the real world. The few non-web-based foot-related simulation courses have focused solely on training for a single skill or "part task" (for example, practicing ingrown toenail procedures on models). This pilot study aimed to primarily investigate the effect of a training program using multiple methods of simulation on participants' clinical confidence in the management of foot ulcers.

**Methods:**

Sixteen podiatrists participated in a two-day Foot Ulcer Simulation Training (FUST) course. The course included pre-requisite web-based learning modules, practicing individual foot ulcer management part tasks (for example, debriding a model foot ulcer), and participating in replicated clinical consultation scenarios (for example, treating a standardised patient (actor) with a model foot ulcer). The primary outcome measure of the course was participants' pre- and post completion of confidence surveys, using a five-point Likert scale (1 = Unacceptable-5 = Proficient). Participants' knowledge, satisfaction and their perception of the relevance and fidelity (realism) of a range of course elements were also investigated. Parametric statistics were used to analyse the data. Pearson's *r *was used for correlation, ANOVA for testing the differences between groups, and a paired-sample t-test to determine the significance between pre- and post-workshop scores. A minimum significance level of *p *< 0.05 was used.

**Results:**

An overall 42% improvement in clinical confidence was observed following completion of FUST (mean scores 3.10 compared to 4.40, *p *< 0.05). The lack of an overall significant change in knowledge scores reflected the participant populations' high baseline knowledge and pre-requisite completion of web-based modules. Satisfaction, relevance and fidelity of all course elements were rated highly.

**Conclusions:**

This pilot study suggests simulation training programs can improve participants' clinical confidence in the management of foot ulcers. The approach has the potential to enhance clinical training in diabetes-related foot complications and chronic wounds in general.

## Background

Foot ulcers are a leading cause of hospitalisation for diabetes-related complications [1]. The vast majority of amputations in the lower limb are preceded by a foot ulcer [1]. In Australia in 2004/05, for example, the management of people with diabetes-related foot ulceration required the use of nearly 130,000 hospital beds and contributed to approximately 3,400 lower extremity amputations and 1,001 deaths [2].

Studies consistently demonstrate that a range of proactive foot ulcer prevention and management strategies can significantly reduce poor diabetes-related foot outcomes [3-10]. Reported outcomes include reductions of amputations (85%) [4], hospitalisation (90%), bed days (90%) [5], costs (85%) [1] and missed worked days (70%) [5]. These multi-faceted strategies include access to multi-disciplinary foot teams, increased use of podiatrists, evidence-based clinical pathways and protocols, and clinical training [3-10].

Clinical training is known to have a beneficial impact on diabetes-related foot ulcer outcomes [3-12]. The authors are not aware of any other clinical training courses that have used multiple forms of simulation training techniques in the management of diabetes-related foot complications and/or chronic wounds in general. Simulation has been defined as a device or environment that attempts to replicate or recreate the real world [13] Simulation training allows the trainer to control the level and complexity of trainee practice and environmental distractions within a safe, controlled learning environment [13]. The development of the Foot Ulcer Simulation Training (FUST) program and this pilot study were seen as a unique opportunity to trial the effectiveness of multiple forms of simulation training in improving clinical confidence in foot ulcer management. It is intended that subsequent follow up studies will aim to investigate longer term impacts on confidence, knowledge, clinical practice and patient outcomes of this program.

Clinician training or continuing medical education (CME) has been described as any way in which clinicians learn after completion of their formal training [14]. A meta-analysis of CME effectiveness revealed a medium effect size in the change in clinician knowledge and attitude, and a smaller effect on clinical practice change and patient outcomes [15]. Importantly, it suggested that larger effect sizes are realised when CME interventions are interactive, use mixed methods, and are in either small groups or groups from a single discipline [15]. It has also been reported that CME should focus on Kirkpatrick's four levels of evaluation: Level I (participant satisfaction), Level II (participant knowledge and attitude change), Level III (participant clinical practice change) and Level IV (patient outcomes) [16].

CME studies evaluating Levels II, III or IV in diabetes-related foot management are limited, and mainly focus on single CME outcome level evaluations. For example, one two-day clinician training package using interactive mixed methods, demonstrated positive effects on Level II outcomes or knowledge and attitude changes in diabetes-related foot management [11]. Another two-day workshop, implemented nation-wide across Brazil, utilised interactive mixed methods and realised positive effects on Level IV outcomes or decreased amputations [12].

Further results of the CME meta-analysis reinforced the need for CME techniques that are innovative, interactive and effective [15]. The literature suggests simulation techniques may fit these future CME needs and outcomes [17].

Patient simulation has been used in the health sector since the 1960s. In the last two decades the use of simulation in both undergraduate and postgraduate medical and nursing training has grown prolifically in the acute or inpatient environments [18-20]. However, simulation training for application in the outpatient environment and amongst allied health disciplines has been a relatively recent development.

The increased uptake of simulation has been driven by several factors including: an increased focus on patient safety; the community's growing lack of acceptance for clinicians to acquire skills on real patients; reduction in direct clinical contact training hours as well as increased patient complexity and demands on healthcare providers [20-25]. Simulation is not designed to replace conventional teaching methods such as lectures, tutorials or experience gained through practical clinical exposure, but to be integrated with established methods to strengthen students' and clinicians' learning experience [25].

The three main principles that form the foundation of simulation are deliberate practice, feedback and debriefing or reflection [25]. Deliberate practice is essential in achieving competency in a particular skill. Simulation provides a safe, controlled environment where participants can develop skills without fear of adverse clinical consequences whilst being supported by prompt expert feedback [17,23,25,26] and encouraged to develop skills in reflective practice [22,27,28].

There are several types of simulation that range from web-based interactive and virtual learning programmes through to full high-fidelity clinical scenario simulation that is reflective of a participant's work environment. The degree to which a simulation replicates reality is called "fidelity" [13]. The extent to which a simulation replicates a real-world system, or is realistic, defines whether they are "high" or "low" fidelity [13]. Each form of simulation has its own uses and learning applications [29]. For this reason, research suggests that simulation courses should aim to incorporate as many different simulation modalities as possible [30]. The combination of part task trainers (often referred to simply as "part tasks") and the use of standardised patients (or referred to as "clinical scenarios") are essential and often under-appreciated as a means of ensuring safe practice and clinical competency [27]. Part tasks are designed to segment complex jobs or activities into their main individual components, for example, practicing endotracheal intubation [13]. Clinical scenarios are designed to simulate an entire complex task, for example the entire emergency management of a motor vehicle accident victim in a simulated emergency room [13].

Research into different training settings and applications has been positive and supportive of simulation [31-34]. Overall, the literature has rated highly simulation's ability to improve participants' technical skills and confidence over the short and long term [31-34]. However, there is a gap in the literature in terms of long-term follow-up investigations into the translation of skills to improve actual clinical practice and patient outcomes [35]. From a preliminary review of recent literature, no studies have yet been able to successfully match course participation with long-term patient outcomes, despite recommendations in the literature [21,36].

The effective use of simulation to improve participants' confidence and acquisition of both technical and non-technical skills suggests that its application to the principles of diabetes-related foot complications or chronic wound care would be advantageous. The use of non-web-based simulation in Podiatry or diabetic foot management has not been widely adopted, except in the utilisation of part tasks for single technical training in basic physical examination, suturing, injection and intravenous techniques, tissue excision, biopsy and ingrown toenail procedures [37]. A review of the literature identified only training in the single technical skill of pressure ulcer classification as an application of simulation training in chronic wound management [38,39].

Moreover, simulation training for application in outpatient settings has rarely been used [40]. Kneebone et al (2007) recommends expanding the application of simulation training to any health professional who performs clinical interventions [17]. This is a way of cementing rudimentary clinical skills that are applied in complex clinical circumstances, as well as in crisis situations [17].

The Foot Ulcer Simulation Training (FUST) course was conceived in 2009 after a Queensland Health 'training needs analysis' survey of podiatrists prioritised the need to train podiatrists practically in high risk foot and foot ulcer management as the most important training need for Queensland Health podiatrists. The course was designed, developed and implemented in 2010 by the Queensland Health Statewide Podiatry Network and Queensland Health Clinical Skills Development Service. The primary aim of this pilot study was to evaluate the impact of a two-day simulation training course on podiatrists' clinical confidence in the management of foot ulcers. Secondary objectives were to determine participants' satisfaction with relevance and fidelity (realism) aspects of the course, and to investigate changes in participants' knowledge.

## Methods

### Setting and participants

The study was located at the Queensland Health Clinical Skills Development Service based at the Royal Brisbane and Women's Hospital in Brisbane, Queensland, Australia. The Clinical Skills Development Service was utilised to help develop and deliver the FUST training course because of their extensive experience in simulation-based training, and their international reputation for innovative programs.

The Medical Research Ethics Committee at the University of Queensland, Australia provided ethical approval for the study. Written informed consent was obtained from all participants prior to commencement of the course and data collection.

The participants in this study were 16 Queensland Health -employed podiatrists who voluntarily attended one of two, two-day FUST courses in May or June 2010. Queensland Health podiatrists were chosen as they are required to prioritise patients with foot ulcers or high risk feet in accordance with the 'Queensland Health Podiatry Services Statement of Core Business' (2009), "Queensland Health podiatrists will deliver evidence based, best practice clinical services for those people with lower limb amputations, ulcerations, peripheral neuropathy, peripheral vascular disease and/or gross foot deformities". Therefore, according to Queensland Health podiatry 'core business', and the aforementioned training needs analysis priority, participation in this training should have been seen as of being a high priority and benefit for all Queensland Health podiatrists. Participation was, however, only open to all base level 'clinician' (Level 3 in Queensland Health Practitioner Award) or 'senior clinician' (Level 4) podiatrists employed by Queensland Health and travel and accommodation was subsidised. An email alert was delivered to all level 3 and 4 Queensland Health -employed podiatrists inviting them to register for the courses. A convenience sample was employed as participants were recruited on a 'first registered, first recruited' basis. The sample of 16 was nearly half of the total eligible level 3 and 4 podiatrists (35) or one third of the total 45 podiatry practitioners employed by Queensland Health. Participants were assigned to one of two course intakes. The first course consisted of eight podiatrists with fewer than three years of clinical experience or predominantly those at level 3. The second group consisted of eight podiatrists with three or more years of clinical experience or predominantly those at level 4. It was assumed that podiatrists with longer clinical experience or level 4 would have had greater experience in the management of diabetes-foot related complications and/or chronic wounds.

The course was developed by an advisory committee of 'specialist clinician' (Level 5) and 'consultant clinician' (Level 6) Queensland Health podiatrists in consultation with endocrinologists and senior simulation co-ordinators. The learning objectives and content were based upon the clinical skills necessary for 'expert assessment and management of existing foot ulcer or lesion' as outlined by The National Minimum Skills Framework for Commissioning of Foot Care Services for People with Diabetes joint report (United Kingdom, 2006) [41]. 'Specialist' and 'consultant' podiatrists, endocrinologists and a senior simulation co-ordinator facilitated the courses. The facilitators were trained in their roles prior to the courses via one day of training and a formal facilitators' manual explaining all aspects of the course in extensive written and pictorial detail. The practical training consisted of orientation to the courses simulation equipment and infrastructure, and practising the facilitation of part tasks, clinical scenarios, debriefing and other facilitation techniques.

### Procedure

Prior to the workshops, all participants were required to ensure completion of a number of pre-requisite interactive web-based or e-learning modules covering theory on the management of all types of foot ulcers, approximately five hours in total. At the beginning of the course, participants were provided with a comprehensive training manual containing learning objectives, learning resources and detailed written and pictorial instructions for each aspect of the course.

The FUST program consisted of two days of practical workshop activities. At least 80% of the course time required participants to participate actively in practical clinical skills or decision-making activities.

The first three sessions of day one consisted of participants practicing foot ulcer management components or part tasks. Participants were required to complete the practice of 22 part task "stations". Each part task station encouraged participants to focus on designated repetitive practice of a particular foot ulcer management component, for example practicing the performance of toe systolic pressures on subjects. Part tasks were categorised into six sections, typically consisting of four 10-15 minute stations per section. Individual stations usually had two participants and one assigned facilitator. The sections consisted of: high risk foot assessment or comprehensive non-invasive neurovascular assessments, foot ulcer assessment, infection management, wound management, off-loading management and multi-disciplinary team work.

The fourth and final session of the first day introduced participants to the "pressure chamber". This consisted of four rooms in which participants worked in pairs on twenty-minute scenario rotations designed so as to integrate the individual skills addressed during the previous part-tasks. Three of the simulated scenarios included a foot model containing a moulage of a foot ulcer, and a manufactured patient medical history. One room was a designated debriefing room with a facilitator present. Participants in the three scenario rooms had the ability to direct any clinical questions to a facilitator observing behind mirrored glass.

The second day consisted of eight simulated scenarios on a 'controlled' range of standardised patients (actors) with simulated foot ulcers and/or other diabetes-related foot complications in a simulated clinical outpatient environment. Additional file 1, Movie file S1 illustrates a short example of a FUST clinical scenario. Two groups of four participants each participated in parallel clinical scenarios throughout the day. In each group participants treated the "patient" in pairs for 25-30 minutes whilst two other participants watched the scenario on live play-back in an adjacent room. During each scenario a facilitator or endocrinologist would observe behind mirrored glass and then enter the room to allow participants to perform a case presentation and to outline their treatment and management plan. As the day progressed the scenarios increased in complexity.

After each scenario a 15-20 minute debriefing session was held with the participants in each group who had either actively participated or observed the scenario. The facilitator was available to provide guidance and offer constructive non-critical feedback, support and expert advice where required.

### Evaluation

**Figure 1:**
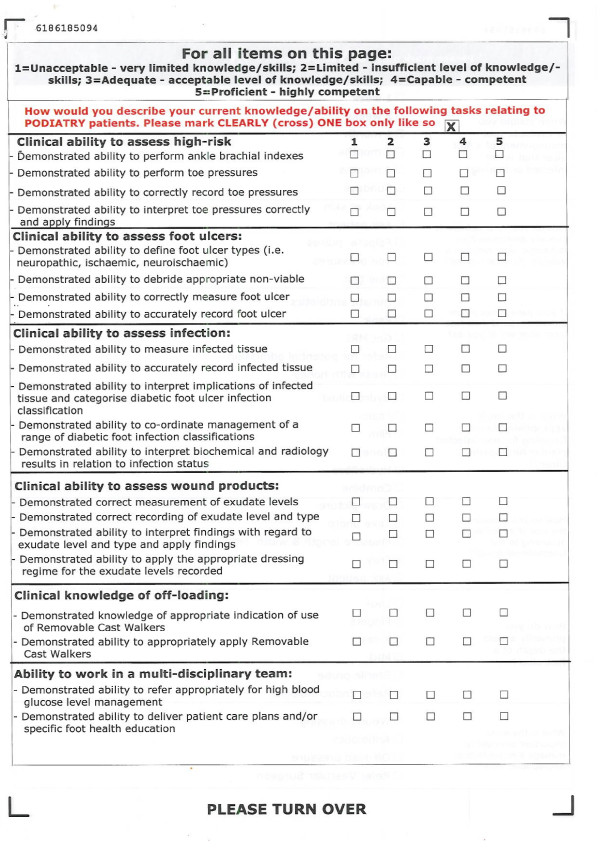
**Clinical confidence surveys**.

**Figure 2:**
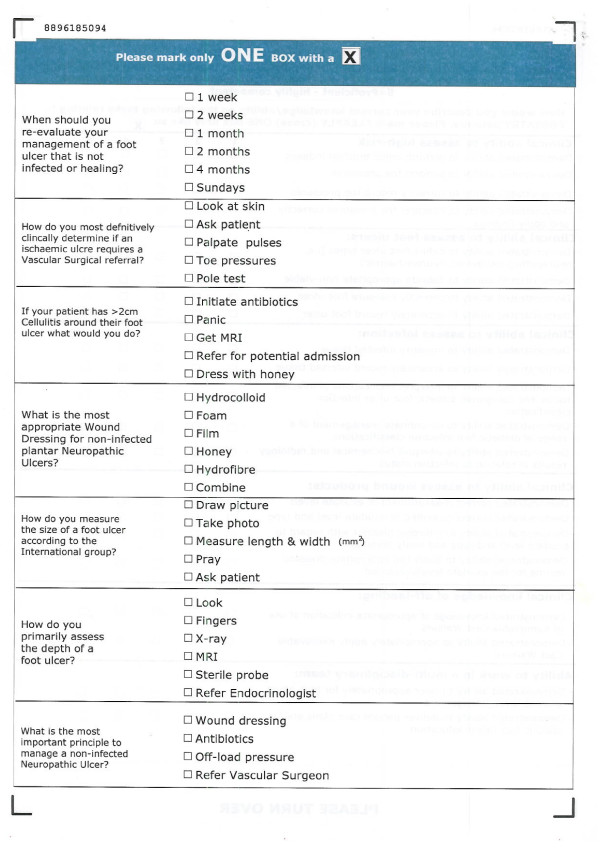
**Clinical knowledge surveys**.

**Figure 3:**
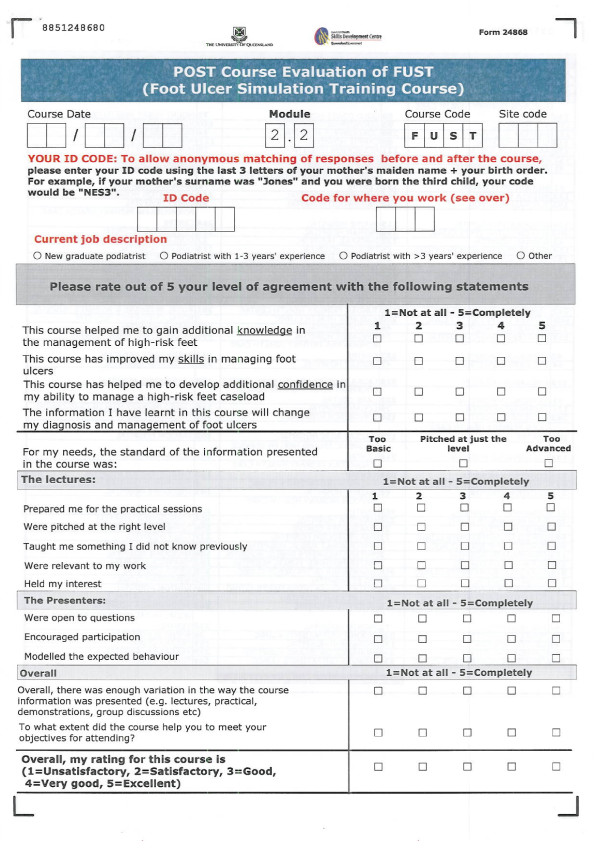
**Satisfaction surveys**.

The overall evaluation of FUST was multi-layered and consistent with Kirkpatrick's four levels of analysis, as recommended for CME [17]. However, this paper will only evaluate short term findings of Levels I and II. It is intended that Levels III and IV will be evaluated in subsequent studies as they require sufficient time to elapse to enable the measurement of outcomes. Evaluation consisted of custom-designed surveys to measure participants' course satisfaction and pre- and post workshop self-rated confidence and knowledge levels in foot ulcer management. The self-rated confidence and knowledge surveys were distributed to, and completed by, participants on the morning immediately prior to commencement of the course and then again at the end of each afternoon and immediately on completion of the course. To ensure anonymity for participants and the matching of responses, a four digit code only understood by each individual participant was used for all evaluations. Participants' clinical confidence was measured across 21 defined foot ulcer management items, this was a subset of the part tasks and scenarios completed over the two-day course, using a five-point Likert scale (1 = Unacceptable-5 = Proficient) (Figure 1). Clinical knowledge was measured across seven multiple choice question items (Figure 2). Satisfaction aspects, including relevance and fidelity were also measured using a five-point Likert scale (1 = Not at all-5 = Completely) (Figure 3).

To gain a more objective view of any change in participants' confidence levels, clinical supervisors from the participants' work place were also asked to assess the participants' confidence or competence. The supervisors were asked to complete the same clinical confidence items and scales as the participants used, with the exception that the supervisors rated the participants according to the extent that they *demonstrated *the skills, whereas the participants rated their level of confidence in them. The supervisors' post workshop survey was not repeated at the conclusion of the FUST course, unlike the participants' survey. It was necessary for the participants to have time to apply the skills they learned at the workshop in their workplace, and for their supervisors to observe and re-assess the participants' competence. It is intended that follow-up supervisors' surveys will be investigated in subsequent studies.

### Statistical analysis

Data were analysed using SPSS 17.0 for Windows (SPSS Inc., Chicago, IL, USA). Although the data were ordinal in nature, the mean score has been reported as well as the median in order to give a more refined interpretation of the results. Parametric statistics were used to analyse the data because there was little difference between the mean and median scores, and significance levels. Pearson's *r *was used for correlation, ANOVA for testing the differences between groups, and a paired-sample t-test to determine the significance between pre- and post workshop scores for confidence and knowledge. The decision to use parametric statistics in the study is supported by recent literature that provides strong evidence of the robustness of parametric statistics when used, *inter alia*, with Likert scales and data with non-normal distributions [42,43]. A minimum significance level of *p *< 0.05 was used throughout.

## Results

All 16 participants had completed the pre-requisite web-based modules. Of the 16 participants who commenced FUST, 15 completed the workshop. One participant in the first group failed to complete the course due to illness unrelated to the FUST course and was unable to complete the post-workshop surveys. The pre-workshop data from the participant that failed to complete the course has been retained in this study.

No statistically significant difference was detected between scores from podiatrists with different levels of experience except on one clinical confidence item and one fidelity item. Podiatrists with more than three years experience reported a greater increased confidence in their ability to refer patients appropriately for hyperglycaemic management, and also greater task fidelity in the off-loading part task than those with less experience.

### Satisfaction

Overall satisfaction with the course was high. Of the 14 out of 15 participants who completed the question on the post workshop survey (one did not record a response to that question), 13 rated the course as being 'excellent' and one as being 'very good'. All participants reported that they had met their objectives for attending FUST 'completely', that the level of the workshop was 'just right', and that the variety in workshop delivery was sufficient.

One hundred percent of participants rated the quality of facilitators as being "excellent" (five out of five for all items). Furthermore, lectures provided during the workshop received a median score of five out of five (mean score range 4.67 - 4.73) on all items including: preparing participants for practical session; being pitched at the right level and relevant to work; holding participants' interest and teaching them something that they did not know previously.

### Relevance and fidelity (realism)

Overall, the mean scores for relevance and fidelity were respectively 4.82 and 4.47 out of 5.

### Clinical knowledge

There were seven knowledge items assessed before and after the workshop. Only one item, 'determining if an ischaemic ulcer requires vascular surgical referral', recorded a statistically significant improvement (*p *= 0.009). Table 1 shows all knowledge items and scores.

**Table 1:** Comparison of pre- and post workshop mean scores for all knowledge items

	*Pre % (n) correct	*Post % (n)* correct
1. Re-evaluation of management of a non-healing, non-infected foot ulcer	14 (87.5%)	14 (92%)
2. Determining if an ischaemic ulcer requires vascular surgical referral	6 (37.5%)	11 (73%)
3. Managing >2cm cellulitis	16 (100%)	15 (100%)
4. Most appropriate dressing for non-infected plantar neuropathic ulcers	15 (94%)	15 (100%)
5. Assessment of the depth of a foot ulcer	16 (100%)	15 (100%)
6. Measurement of foot ulcer according to the International group	14 (87.5%)	15 (100%)
7. Management principle of non-infected neuropathic ulcer	16 (100%)	15 (100%)

*Only 15 post workshop evaluations were received compared to 16 pre-workshop evaluations

### Clinical confidence

**Figure 4:**
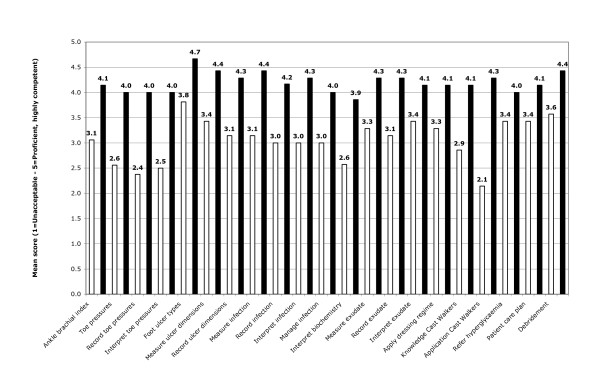
**Clinical confidence comparison of pre- and post- mean scores**. * White bars = Pre-workshop scores. # Black bars = Post-workshop scores.

Participants' clinical confidence was observed to have improved 42% overall between pre- and post-completion of FUST, with respective mean scores of 3.10 compared to 4.40 (*p *< 0.05). Figure 4 demonstrates the statistically significant (*p *< 0.05) improvement in participants' confidence levels across all 21 clinical items. Improvements ranged from 17% for ability to refer for hyperglycaemia management, to 100% for ability to apply a Removable Cast Walker. Additionally, Table 2 shows that regardless of their level of experience, all groups had a similar statistically significant improvement in their confidence levels following the course (*p *< 0.05).

**Table 2:** Comparisons of overall pre- and post workshop scores for confidence by years of clinical experience

	Pre-FUST	Post-FUST
New Graduate	3.1 (SD 0.29)	4.2 (SD 0.33)
1 - 3 years' experience	3.0 (SD 0.13)	4.2 (SD 0.49)
>3 years' experience	3.2 (SD 0.57)	4.6 (SD 0.30)

SD. = Standard deviation

Ten participants had supervisors who completed and returned the parallel supervisors' survey of participants' confidence levels across the twenty-one items. The other five participants did not have a podiatry clinical supervisor, and therefore, could not be rated by a supervisor. There were statistically significant differences (*p *< 0.05) in the scores for only six of the twenty-one items which were: definition of foot ulcer types; appropriate debridement of non-viable tissue; correct measurement of foot ulcer dimensions; measurement of infected tissue; accurate recording of infected tissue; interpretation and classification of infected tissue.

## Discussion

The majority of published studies have focused on simulation training's impact in an emergency, trauma or surgical environment [31-35,40,44-46]. This study was unique in that it suggests improved clinical confidence of participants after using simulation training techniques related to the management of diabetes-related foot complications and/or, chronic wounds, in this case foot ulcers. The success of this pilot study supports suggestions that simulation is flexible enough to lend itself to multiple clinical training environments, disciplines and needs [21,26,47-49]. Additional advantages of simulation training in healthcare include its ability to allow participants the opportunity to develop, practice and integrate technical and non-technical skills [21,27,29,47,48].

The developers of the FUST course adopted a mixed method course design, as described and recommended by other best-practice CME programmes [15], and applied them to clinical training in outpatient diabetes-related foot complications and chronic wounds. These CME principles included the use of interaction (at least 80% of the time) and mixed methods (case studies, numerous low-fidelity part tasks, high-fidelity full clinical scenarios, and regular non-judgemental debriefing exercises) in small single-discipline groups (of eight podiatrists per course) [15]. FUST also incorporated the simulation principles of deliberate practice, feedback and debriefing [25].

The FUST course avoided the common mistake of some simulation programmes of directly replacing conventional teaching methods with simulation techniques [25]. Completion of web-based learning modules was a pre-requisite to the workshop and provided the conventional theoretical foundation for the practical two-day FUST course. Brief lectures were also integrated into the workshop to summarise the theory before practical interactive tasks were commenced.

Participants' overall satisfaction was high and reflected the course's integration of best practice CME and simulation principles. Participants had their learning needs met completely, and importantly, felt the variety in course delivery was sufficient and pitched at just the right level.

The importance of appropriate training in the facilitation of FUST was evidenced in the participants' positive rating of the facilitators who provided a safe and non-judgemental environment where participants could practice new techniques and receive timely and structured feedback [21,26,29,47-49]. All participants reported that the facilitators had created an environment where attendees were encouraged to participate, ask questions, and where the facilitators had demonstrated the expected behaviour. Additionally, a number of participants suggested that participating in the course "was fun" which is in line with adult learning principles that "fun and enjoyable" training enhances the effectiveness of learning [50].

Deficits in realism and fidelity are commonly reported limitations with manikins and the use of actors in standardised patient scenarios who lack the clinical knowledge to accurately reflect a clinical situation [18,27,29,49]. However, it is notable that the participants in this study rated highly the relevance and fidelity of their interactions with the eight clinical scenarios. This may be partially attributed to the use of experienced clinicians to act as patients in clinical scenarios, as well as the realistic look and feel of the foot models. Arguably, the clinician actors were able to provide more flexible and realistic clinical responses than those confined to a predetermined script. The perceived high level of relevance and fidelity suggest that FUST meets the CME criteria for innovation and interactivity [15]. The formal curriculum, learning objectives, detailed instruction manuals, practical training of facilitators, and the use of standardised part task trainers, and a range of standard clinical scenarios should ensure the standardised high quality delivery of FUST in most clinical training environments.

Simulation training in healthcare is consistently rated by participants as a highly effective and enjoyable education medium [48,51]. The FUST course was no exception. Although, this appears to indicate a successful course on its own, the literature suggests that Level I CME ratings are a poor indicator of clinical effect. Direct analysis of Level II clinical knowledge, attitudes and skills at least is required to determine the impact on clinical practice and patient outcomes [15].

Minimal improvement was recorded in clinical knowledge as pre-course test scores were already high. This "ceiling effect" (when scores are close to the highest they can be) [52] was somewhat expected given that participants' had existing high levels of clinical involvement and interest in the area and the pre-requisite completion of learning theory via web-based modules in the months prior to attending the course. However, the course should have served to reinforce the participants' learning from the detailed manual and the learning resources provided.

All participants' confidence levels rose significantly in all the areas covered by FUST, regardless of their years of podiatry experience. One may infer from these results that doing a workshop such as FUST is worthwhile even for experienced podiatrists, as it provides the opportunity to refresh skills and consolidate a clinician's understanding of foot ulcer management. This particular confidence improvement was only measured over the short term. However, other simulation studies have demonstrated longer term confidence retention following short-term confidence improvements compared to conventional training [48].

The supervisors' assessment of the participants' pre-FUST competence in the skills covered by the workshop aligned with participants' own confidence ratings. Supervisors' results indicate that the collective participants' pre-test or baseline confidence or competence was only adequate, rather than competent or proficient. Similarities in the ratings provided by participants and supervisors indicate that participant ratings were relatively objective and not unduly affected by self-report bias. Subsequent long-term follow up of both participants and supervisors, in future research, will provide a clearer picture.

A large body of evidence exists in support of simulation's ability to increase participants' confidence [53]. Increased confidence levels have been associated with self-efficacy and higher rates of participants actively seeking opportunities to further develop newly acquired skills [36,53]. Self-efficacy is an important outcome from any training program as it reflects participants' ability to translate acquired skills into day-to-day clinical practice [36,54]. Evaluating participant confidence levels is also consistent with Kirkpatrick's four levels of evaluation, and supported the rationale behind its inclusion in this pilot study [16].

Three potentially significant methodological limitations existed in this study. Firstly, the sample size was small. However, with the promising results of this pilot study it can be recommended that larger studies with greater numbers be undertaken.

A second limitation was the absence of a matched control group. This was partially addressed, by using matched participant and supervisor pre-workshop scores as a baseline comparator. It is recommended in future larger studies that a control group is included. Furthermore, this serves to highlight another limitation of potential investigator bias; five of the ten returned pre-intervention supervisor surveys were from supervisors who were either investigators or facilitators of the impending FUST course. This limitation is likely to have been minimised as the study's information sheet recommended supervisors and participants use the supervisors' ratings as part of their participants' annual formal Queensland Health 'Performance Appraisal' to maximise objectivity of this item from supervisors.

Thirdly, performing the pre-knowledge test after the theoretical web modules were completed may have been a limitation. The literature strongly suggests the need for conventional lectures as a theoretical foundation to complement the simulation practice [25]. It was decided to use the existing web-based professional development modules already developed for Queensland Health clinicians as the conventional lecture component. These modules had been recommended to Queensland Health podiatrists as a professional development component of their performance appraisals for at least 12 months prior to the conception of this study. Thus, the imparting of this knowledge was unable to be controlled in this study. Other simulation studies have also found the timing of pre-knowledge tests to fit conventional lectures challenging, and have followed similar methodology to FUST in this regard [48].

Other perceived limitations of this study included potential bias in recruiting subjects with a low level of high risk foot knowledge and clinical confidence because this may have over inflated any effect size. The investigators believe this limitation was minimised by the selection of participants that work predominantly with patients with diabetes-related foot complication and chronic wounds as per the aforementioned Queensland Health Podiatry Services Statement of Core Business (2009). However, again with the promising results of this pilot study's impact on participants with sound existing levels of high risk foot confidence and knowledge, further studies investigating the impact on participants with low levels of existing high risk foot knowledge and clinical confidence would be recommended.

Simulation training is highly facilitator-intensive and its cost is a commonly cited disadvantage [27,29,48,49]. Cost-benefit analyses of simulation programs are needed to justify their expense in terms of improved clinical performance and patient outcomes. Another barrier to wider implementation is the lack of evidence to support the translation of simulation-acquired skills into actual clinical practice and improved patient outcomes [27,29,48,49]. Reasons for this shortfall in research include the difficulty of establishing causality and related methodological issues such as obtaining sufficiently large sample sizes for long-term follow up [26,36].

## Conclusion

FUST is the first pilot study to investigate the use of mixed modality simulation training techniques in the management of diabetes-related foot complications and/or chronic wounds. The FUST study has shown proof of concept for the use of simulation in foot ulcer management training. It supports the commonly-cited hypothesis that simulation is effective in generating participants' interest whilst facilitating repetitive and reflective practice. The study has demonstrated the potential to improve clinicians' confidence, knowledge and satisfaction in the management of foot ulcers through an integrated simulation-based training program. Clinical training literature suggests increased clinical self-confidence contributes positively to improved patient outcomes. Larger prospective studies using foot ulcer simulation clinical training programs are recommended to investigate participants' confidence, knowledge, clinical practice and patient outcomes, such as hospitalisation and amputation rates.

## List of Abbreviations

CME: Continuing Medical Education; FUST: Foot Ulcer Simulation Training.

## Competing interests

The authors declare that they have no competing interests.

## Authors' contributions

PAL conceived, designed, researched data, contributed to discussion, wrote and reviewed/edited the manuscript. ELM researched data, contributed to discussion, wrote and reviewed/edited the manuscript. PMR designed, researched data, contributed to discussion and reviewed/edited the manuscript. FMB contributed to discussion, wrote and reviewed/edited the manuscript. SJ, EMK and GMP designed and reviewed/edited the manuscript. MCK conceived and reviewed/edited the manuscript. All authors have read and approved the final manuscript.

## Supplementary Material

Additional file 1 **Movie file S1 - Example of a portion of a FUST Clinical Scenario**. Click here for file
